# Negative Association Between Allopregnanolone and Cerebral Serotonin Transporter Binding in Healthy Women of Fertile Age

**DOI:** 10.3389/fpsyg.2018.02767

**Published:** 2019-01-11

**Authors:** Inger Sundström Poromaa, Erika Comasco, Torbjörn Bäckström, Marie Bixo, Peter Jensen, Vibe G. Frokjaer

**Affiliations:** ^1^Department of Women’s and Children’s Health, Uppsala University, Uppsala, Sweden; ^2^Science for Life Laboratory, Department of Neuroscience, Uppsala University, Uppsala, Sweden; ^3^Department of Clinical Sciences, Obstetrics and Gynecology, Umeå University, Umeå, Sweden; ^4^Neurobiology Research Unit and Center for Integrated Molecular Brain Imaging, Copenhagen University Hospital Rigshospitalet, Copenhagen, Denmark; ^5^Mental Health Services Copenhagen, Copenhagen University Hospital Rigshospitalet, Copenhagen, Denmark

**Keywords:** allopregnanolone, brain, mood, PET, serotonin transporter, women, 5HTT

## Abstract

Allopregnanolone is a metabolite of the sex hormone progesterone, with suggested relevance for female mood disorders. While allopregnanolone and serotonin are known to influence psychological well-being, the molecular and psychological specifics of their relationship are to date poorly understood, especially in women of fertile age who experience regular fluctuations of progesterone across the menstrual cycle. Availability of serotonin in the synaptic cleft is regulated by the serotonin transporter (SERT), which can be imaged in the living human brain by use of positron emission tomography (PET) and the radiotracer [^11^C]DASB. To evaluate sex-specific allopregnanolone-SERT interactions, the present study investigated the relationship between cerebral SERT availability, serum allopregnanolone levels and psychological well-being in women of fertile age. Brain imaging data, self-reported symptoms of mental distress and emotion regulation, and biobank material from ninety healthy women were available from the Center for Integrated Molecular Brain Imaging (CIMBI) database. Age, BMI, and daylight minutes were included as covariates in the analyses and SERT genotype (5-HTTLPR) was considered a potential confounder. Lower serum allopregnanolone levels were associated with higher SERT binding in the prefrontal cortex. Moreover, allopregnanolone levels were negatively associated with measures of alertness, although this finding was not mediated by prefrontal cortex SERT binding. These findings suggest a link between the typical psychological well-being experienced in the follicular phase when allopregnanolone levels are low and higher SERT in the prefrontal cortex, a region for higher cognitive functions and top-down regulation of emotions.

## Introduction

Progesterone is, together with estradiol, one of the ovarian steroid hormones. Progesterone fluctuations are pronounced during the menstrual cycle, but even more extreme variations are noted across pregnancy and postpartum. Increasing evidence suggest that progesterone influences emotion processing in healthy, naturally cycling women (Toffoletto et al., 2014; Comasco and Sundstrom-Poromaa, 2015). So far, earlier work supports worsened emotion recognition, enhanced emotional memory and increased amygdala reactivity at times of luteal phase progesterone levels (van Wingen et al., 2008; Sundstrom Poromaa and Gingnell, 2014). Furthermore, progesterone has been implicated as a causal agent for premenstrual syndrome and premenstrual dysphoric disorder (PMDD) (Sundstrom et al., 1999; Segebladh et al., 2009). However, while it may be assumed that some of these effects are mediated via the progesterone receptor, some of the typical progesterone-induced mood symptoms could equally well be mediated by γ-aminobutyric acid (GABA)-active progesterone metabolites, such as allopregnanolone (Backstrom et al., 2012). Of relevance, allopregnanolone serum concentrations temporarily follow that of progesterone during the menstrual cycle, but with an off-set of one to two days (Ottander et al., 2005). Further, whereas progesterone levels increase by 25-fold during the luteal phase, allopregnanolone levels vary by approximately four-fold (Nyberg et al., 2007).

Animal models point to sedative, anxiolytic, anti-convulsant, and neuroprotective properties of allopregnanolone (Kask et al., 2008; Melcangi et al., 2011; Brunton, 2015). In humans, a distinct role of allopregnanolone for female mood disorders has been difficult to delineate, and at present findings point in two directions. First, in pregnant women low levels of allopregnanolone have been associated with depressive symptoms (Hellgren et al., 2014, 2017), and a proof-of-concept study recently demonstrated that allopregnanolone infusion in the postpartum period was able to rapidly alleviate postpartum depressive symptoms (Kanes et al., 2017). On the other hand, in terms of the menstrual cycle-induced mental health problems, the allopregnanolone antagonist sepranolone was recently demonstrated to hold promise as treatment of PMDD (Bixo et al., 2017). These disparities in effect may be explained by differences in underlying pathophysiology, but potentially also by a suggested inverted U-shaped relationship between allopregnanolone and psychological wellbeing (Backstrom et al., 2014). The relationship between allopregnanolone and serotonergic neurotransmission has received little attention in humans, but animal studies have shown that acute administration of fluoxetine increases allopregnanolone levels in the brain of female rats (Fry et al., 2014; Devall et al., 2015) and diminishes their sensitivity to stress (Devall et al., 2015). Clearly, further studies are needed to elucidate the role of allopregnanolone in female mood disorders, and its’ relationship with the serotonin system.

Recently, menstrual cycle phase-dependent associations between allopregnanolone and fMRI resting state functional connectivity was demonstrated in healthy women (Syan et al., 2017). In the mid-follicular phase, allopregnanolone levels correlated negatively with resting state functional connectivity between the seed region of the default mode network, the posterior cingulate cortex, and the somatosensory association cortex in the sensorimotor network. In the late luteal phase, among other associations, allopregnanolone levels correlated negatively with connectivity between another region of the default mode network, the medial prefrontal cortex (mPFC), and primary and association visual cortices, but positively with connectivity between the mPFC and the entorhinal cortex in the limbic system (Syan et al., 2017). Moreover, a one-subject longitudinal study found progesterone-dependent modulation of resting state connectivity between the hippocampus and dorsolateral prefrontal cortex (Arelin et al., 2015). Thus, the primary brain regions of choice for this study were the midbrain, pallidostriatum and prefrontal cortex, all important representatives of serotonergic neurotransmission, and amygdala, insula, posterior cingulate and hippocampus for their association to emotion processing and allopregnanolone or progesterone-influenced connectivity.

In terms of molecular imaging of markers of serotonergic neurotransmission, no study to our knowledge has investigated serotonin transporter (SERT) binding in relation to allopregnanolone. Thus, the present study sought to investigate cerebral SERT binding in relation to allopregnanolone serum levels in women of childbearing age. Allopregnanolone serum concentration was expected to negatively associate with SERT availability, and also to influence measures of mood.

## Materials and Methods

### Subjects

Healthy women (age < 50 years) with regular menstrual cycle (23–35 days), BMI < 50 kg/m^2^, and no use of hormonal contraception or therapy, who had undergone PET scanning on a high resolution research tomograph (HRRT) and had a serum sample from the day of the PET scan stored in the CIMBI Biobank (Knudsen et al., 2016) were included in the study. With these criteria, serum samples of 92 women who had undergone [^11^C]DASB scan were available for the study. Two blood samples could not be analyzed for technical reasons, leaving a study population of 90 healthy women for the [^11^C]DASB analyses.

Besides exclusion criteria for neuroimaging, we excluded subjects with clinically relevant medical history, such as neurological or psychiatric disorders, and history of severe head trauma, drug or alcohol abuse, or clinically relevant findings on routine blood chemistry were excluded. All participants had normal findings on brain magnetic resonance imaging (MRI) and displayed no psychopathology according to the revised symptom checklist (SCL-90-R) (Derogatis and Savitz, 1999) and the major depression inventory (MDI). The participants have previously been included as healthy volunteers in studies on GnRH agonist treatment (Frokjaer et al., 2015; Macoveanu et al., 2016; Fisher et al., 2017a), serotonin transporter binding (Fisher et al., 2017b), gastric bypass surgery (Haahr et al., 2015), and functional connectivity (Beliveau et al., 2015). All projects were approved by the Copenhagen Region Ethics Committee (KF-01-2006-20, KF-01-124/04, H-1-2010-085, and H-2-2010-108), and the women provided written informed consent for participation.

### Allopregnanolone Analyses

Blood samples were collected on the day of the PET scan. The blood samples were centrifuged immediately after the phlebotomy and plasma was stored at -20°C. Allopregnanolone plasma concentrations were determined by Umeå Neurosteroid Research Center, in one batch, as previously described (Timby et al., 2006). For measurement of allopregnanolone, we used radioimmunoassay (RIA) after a first step involving extraction with diethyl ether and purification by celite chromatography, the latter to reduce cross-reactivity. The antibody used in the RIA had been raised against 3α-hydroxy-20-oxo-5α-pregnan-11-yl carboxymethyl ether coupled with bovine serum albumin (AgriSera AB, Umeå, Sweden). A RackBeta (Wallace, Finland) scintillation counter was used to count the samples. The allopregnanolone detection limit was 25 pg/ml, with intraassay coefficient of variation for of 6.5% and an interassay coefficient of variation of 8.5%.

### SERT Imaging

SERT binding was imaged with [^11^C]DASB-PET during a 90- min dynamic acquisition directly following the bolus injection of the tracer. A Siemens ECAT HRRT scanner (Siemens, Munich, Germany), operating in three-dimensional acquisition mode, with an in-plane resolution of 2 mm, was used to obtain the PET scans. The outcome of the [^11^C]DASB binding was the ratio between specific binding and non-displaceable binding of the tracer. We used a modified reference tissue model, using cerebellum as a reference region (the vermis part excluded), designed to quantify [^11^C]DASB (multilinear reference tissue model/multilinear reference tissue model 2) (Ichise et al., 2003), and implemented in PMOD (version 2.9; PMOD Technologies, Zurich, Switzerland). Movement correction was performed by AIR (Woods et al., 1992). The co-registration of the [^11^C]DASB mean image to the high-resolution T1-weighted MR image was done using SPM8 (Ashburner and Friston, 1997). The exact details on the [^11^C] DASB imaging has been presented previously (Frokjaer et al., 2009).

### Volumes of Interest

Volumes of interest for this study were outlined on the participant’s MRIs, as described previously (Svarer et al., 2005). In order to constrain the number of statistical comparisons we pooled a number of regions. This approach was based on the observation of high correlation of SERT binding between cortical and high-binding subcortical regions (Erritzoe et al., 2010). Thus, an average SERT binding potential was computed for each participant for the prefrontal cortex (computed by pooling orbito-, superior-, and medial- and inferior-frontal cortex), the pallidostriatum (a combined subcortical region), and the midbrain (including the raphe nuclei). These three VOIs were the primary outcomes of the study. In addition, four regions served as secondary VOIs; amygdala, insula, hippocampus, and posterior cingulate cortex. We chose these VOIs as SERT binding in these regions is relevant in relation to previously described allopregnanolone actions (Arelin et al., 2015; Syan et al., 2017). Time-activity curves from the VOIs and cerebellum (nonspecific binding) were only obtained from gray matter voxels, except in the midbrain, where separation of gray and white matter is difficult.

### Mood, and Alertness

Emotional functioning and mood symptoms were assessed using the following questionnaires: Profile of Mood States (POMS); Cohen’s Perceived Stress Scale (PSS); Symptom Checklist-revised (SCL); and Global Severity Index of SLC score (SCL-GSI). A Simple Reaction Time task was performed to measure general alertness and motor speed, as described in (Stenbaek et al., 2016).

### 5-HTTLPR

The serotonin transporter-linked polymorphic region (5-HTTLPR) (Heils et al., 1996; Lesch et al., 1996) was genotyped in 87 of the participating women, as described in (Kalbitzer et al., 2010). Genotype frequencies were in Hardy-Weinberg equilibrium; SS = 22.2%, SL = 41.1%, and LL = 33.3%).

### Statistics

The association between allopregnanolone serum concentrations and SERT binding was evaluated by multiple linear regression models. Previous findings have indicated that cerebral SERT binding is influenced by season, age (declining with increasing age), and body mass index (BMI) (Kalbitzer et al., 2010; Tyrer et al., 2016). For these reasons, the weighted least square regression models of SERT binding included age, BMI, and daylight minutes as covariates, all entered as continuous variables. Because allopregnanolone levels were not normally distributed, we also weighted the regression models against the rank order of allopregnanolone. Further all models were carefully checked to ensure that unstandardized residuals were normally distributed. Finally, we validated the weighted least square regression models by use of robust linear regression using an MM estimator.

Path analysis, with bootstrapping to yield confidence intervals for the indirect effect, was performed to evaluate if the effect of allopregnanolone on simple reaction latency was mediated by prefrontal cortex SERT binding. Statistical analyses were performed by SPSS 24.0, SPSS Amos 24.0, and R 3.4.3 using the RobustBase 0.92 package.

## Results

As described in Table 1, women were on average young adults, slightly over-weight and healthy from a psychiatric perspective. According to normative allopregnanolone levels (Nyberg et al., 2007), the majority of women was assessed in the follicular phase, 82/90 (91.1%). The highest SERT binding levels on average were found in the midbrain and pallidostriatum, whereas the lowest levels were noted in the prefrontal cortex and posterior cingulate (Table 1).

**Table 1:** Demographic data, allopregnanolone levels, and [^11^C]DASB binding in the brain.

	*n* = 90	
	
	Mean ± SD	Range
Age, years	27.4 ± 8.2	18–49
BMI, kg/m^2^	25.4 ± 6.3	17.0–43.9
Allopregnanolone, nmol/l	0.61 ± 0.50	0.13–3.70
Estradiol, pmol/l^a^	203 ± 130	60–810
Progesterone, nmol/l^a^	0.2 ± 0.13	0.06–0.8
Daylight, minutes	739 ± 201	426–1052
SCL global severity index	0.3 ± 0.2	0.01–1.16
Total Mood Disturbance score	5.7 ± 20.5	-21–86
**[^11^C]DASB BP_ND_**		
Prefrontal cortex	0.38 ± 0.09	0.12–0.61
Midbrain	2.06 ± 0.29	1.22–2.71
Pallidostriatum	1.98 ± 0.35	1.12–3.02
Total amygdala	1.78 ± 0.35	0.82–3.04
Total insula	0.89 ± 0.14	0.42–1.32
Total hippocampus	0.71 ± 0.13	0.33–1.09
Total posterior cingulate	0.49 ± 0.10	0.16–0.69
Injected dose, MBq	571 ± 56	360–642
Injected mass per kilo, μg/kg	0.03 ± 0.04	0.00–0.24
Specific activity, GBq/mmol	177 ± 136	10.0–675

*^a^Estradiol and progesterone serum concentrations only available in 72 women. SCL-GSI, Global Severity Index of the Symptom Checklist-revised score.*

**FIGURE 1:**
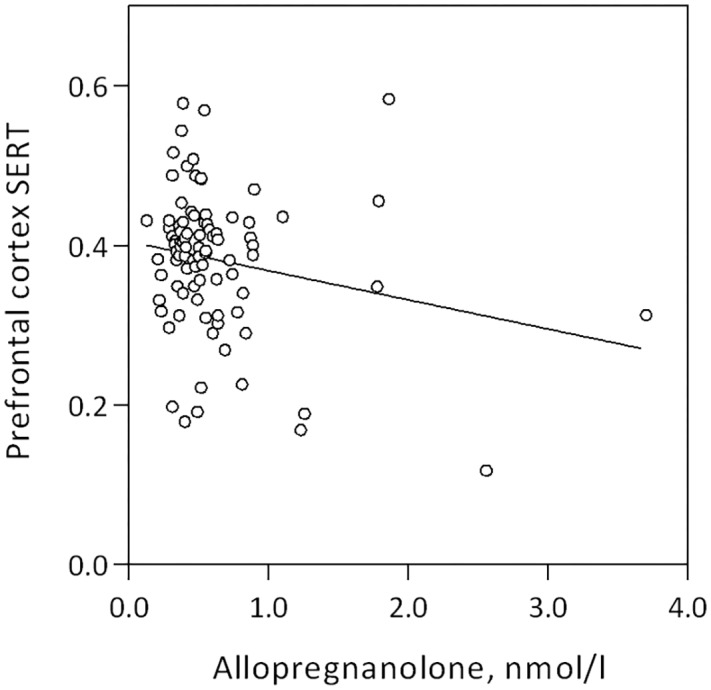
Linear association between prefrontal cortex SERT binding and serum allopregnanolone level in women of fertile age, slope = –0.038, *SD* = 0.017, β = –0.248, *p* = 0.024.

Serum allopregnanolone levels were negatively associated with SERT binding in the prefrontal cortex, pallidostriatum, insula, posterior cingulate, and hippocampus (Table 2 and Figure 1). Overall, the models, which also included age, BMI and daylight minutes, explained 6–23% of the variance in SERT binding levels across regions of interest, with the highest explanatory value in the midbrain, and the lowest in the prefrontal cortex (prefrontal cortex: (slope = -0.038, *SD* = 0.017, β = -0.248, *p* = 0.024) (Table 2). However, the only allopregnanolone association that survived robust regression was the one with SERT binding in the prefrontal cortex (slope = -0.011, *SD* = 0.003, β = -0.196, *p* < 0.001), whereas the robust regressions between allopregnanolone and SERT binding in the pallidostriatum and hippocampus were in borderline significance (pallidostriatum; slope = -0.162, *SD* = 0.083, β = -0.236, *p* = 0.054, hippocampus; slope = -0.049, *SD* = 0.027, β = -0.187, *p* = 0.071). Further, the negative association between allopregnanolone and prefrontal cortex SERT binding remained when also adjusted for estradiol levels (slope = -0.092, *SD* = 0.029, β = -0.359, *p* = 0.002, *n* = 70). Considering 5-HTTLPR genotype, as a proxy of SERT functionality, did not influence any of the above mentioned findings (tested both as first order effects and interactions).

**Table 2:** Multiple linear regression analyses on allopregnanolone influence on [^11^C]DASB binding.

	Slope				Adjusted
	estimate	SD	β	*p_adj_*	R^2^
Prefrontal cortex	-0.038	0.017	-0.248	0.024	0.06
Midbrain	-0.070	0.048	-0.149	0.147	0.16
Pallidostriatum	-0.172	0.006	-0.302	0.005	0.10
Total amygdala	-0.116	0.062	-0.146	0.067	0.06
Total insula	-0.072	0.025	-0.300	0.005	0.11
Total hippocampus	-0.062	0.022	-0.293	0.006	0.13
Total posterior cingulate	-0.044	0.016	-0.264	0.008	0.23


*Models adjusted for age, BMI, daylight minutes on the scanning day, and weighted against rank order of allopregnanolone levels.*

**FIGURE 2:**
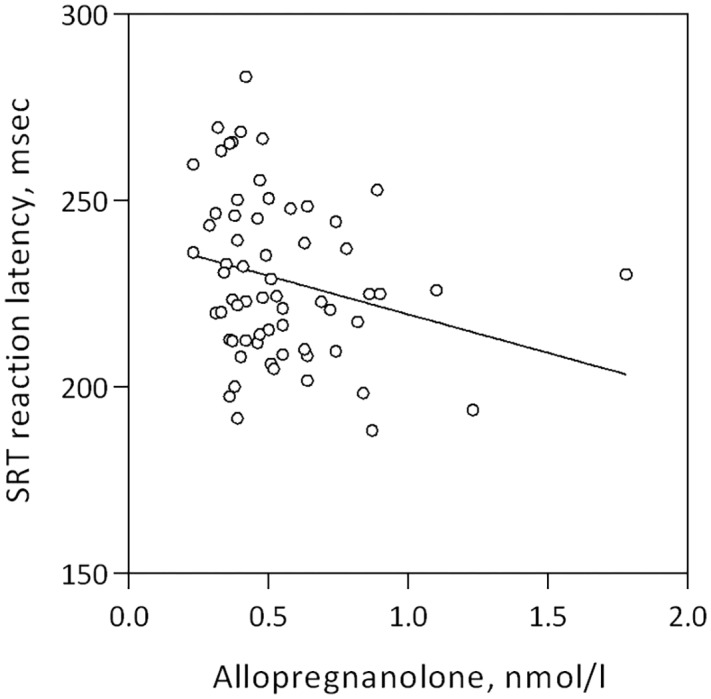
Linear association between mean reaction latency in the simple reaction test and allopregnanolone levels in women of fertile age, Spearman’s rho = –0.267, *p* = 0.033.

Allopregnanolone levels were negatively correlated with mean reaction latency and percent correct trials in the Simple Reaction Test, Table 3 and Figure 2. Women who reacted slower displayed higher allopregnanolone levels, and similarly, made more errors (Table 3). The path analysis revealed that allopregnanolone exerted a significant direct effect on frontal cortex SERT binding (standardized estimate -0.321, *p* = 0.034) and a borderline significant effect on reaction latency (standardized estimate -0.179, *p* = 0.061). However, the indirect effect of allopregnanolone on reaction latency, mediated by prefrontal cortex SERT binding in the path model, was insignificant (standardized estimate -0.066, *p* = 0.061). Allopregnanolone serum concentrations did not influence any of the emotion- or stress-related measures (Table 3).

**Table 3:** Correlations between allopregnanolone and mood proxies.

	*n*	Spearman Rho	*p*
POMS Total Mood Disturbance (TMD) score	75	0.176	0.132
SCL-GSI	90	0.136	0.202
Cohen PSS	89	0.015	0.891
**SRT total mean reaction latency**	**64**	-0.267	**0.033**
**SRT percent correct trials**	**64**	-**0**.**309**	**0.013**

*POMS, Profile of Mood States; PSS, Perceived Stress Scale; SCL, Symptom Checklist-revised; SCL-GSI, Global Severity Index of SLC score; SRT, Simple Reaction Test; TMD, Total Mood Disturbance. SRT, Simple Reaction Task.*

## Discussion

Mapping of the allopregnanolone-serotonin transporter relationship in women of fertile age demonstrated a negative correlation between peripheral levels of this neuroactive steroid and prefrontal cortex availability of the protein responsible for serotonin re-uptake from the synaptic cleft. The major limitations of this study was that measurements were mostly performed in the follicular phase, and that the study only included healthy women.

Allopregnanolone, produced both by the ovaries and in the brain, acts not only as a transcription regulator but also as a GABA agonist by binding to its site on the GABA receptor A (GABA_A_). Allopregnanolone binding to the GABA_A_ receptor leads to enhanced chloride ion flow and decreased GABA unbinding, which is reflected by increased frequency of inhibitory postsynaptic current and desensitization of the receptor (Wang, 2011). With GABA being the major inhibitory neurotransmitter in the brain, present in one third of all synapses, the effects of allopregnanolone are expected to be widely spread, albeit with some differences, depending on the localization and subunits of the GABA_A_ receptor (Wang, 2011). As the women in this study were assessed at a time-point when progesterone and allopregnanolone levels are low, the present findings may reflect interactions between SERT and GABA-mediated tonic conductance at the extra-synaptic level, via α_4_β_2_δ GABA receptors (Wang, 2011). Presence of GABA_A_ receptors outside the synaptic cleft has been shown in the hippocampal formation and cortex (Wang, 2011), brain regions expressing SERT. Additionally, phasic modulation of neuronal excitability by GABA can be also influenced by synaptic GABA_A_ receptors found ubiquitously in the brain (Wang, 2011). Clearly, longitudinal investigations will be needed to understand the relationship between serotonergic neurotransmission and fluctuations of allopregnanolone and mood across the menstrual cycle.

The present findings highlight a relationship between allopregnanolone and SERT, particularly in the prefrontal cortex. In this key region for higher cognitive functions and top-down regulation of emotions, GABA as well as serotonergic projections are widespread (Mengod et al., 2015). Some evidence on GABA-serotonin reciprocal modulation may be derived from animal studies (Svensson et al., 2000; Yan, 2002), but human data is scarce. In terms of molecular imaging of markers of serotonergic neurotransmission in relation to progesterone, 5-HT_1A_ receptor binding was negatively correlated with progesterone at the whole brain level in postmenopausal women (Stein et al., 2014), whereas combined estrogen-progesterone treatment in postmenopausal women was associated with widespread increased cortical 5-HT_2A_ receptor binding (Moses et al., 2000; Moses-Kolko et al., 2003). However, binding of these two receptors did not correlate with progesterone in another study of pre- and post-menopausal women (Moses-Kolko et al., 2011). To our knowledge, this is the first study investigating SERT binding in relation to the progesterone metabolite allopregnanolone in women of fertile age.

Higher SERT in the presence of low allopregnanolone levels, and independently of estradiol levels, may explain psychological well-being during the follicular phase of the menstrual cycle. In fact, no association of allopregnanolone with mood and stress was found in this group of healthy women, likely because of the healthy state and the limited variation in these measures during the follicular phase. Previously, lower SERT binding has been associated with depression (Parsey et al., 2006; Newberg et al., 2012). Dysregulation of both allopregnanolone and serotonin have been implicated in anxiety, irritability aggressiveness, as well as cognitive impairment (Schule et al., 2014); however, knowledge of their interaction at the molecular level is scarce. While enhancing effects of SSRIs on peripheral allopregnanolone levels have been demonstrated (Uzunov et al., 1996; Griffin and Mellon, 1999; Pinna et al., 2009), effects in the other direction remain to be studied. Additionally, neurotrophic-like functions have been demonstrated for both allopregnanolone and serotonin, though more limited to developmental stages for serotonin (Whitaker-Azmitia, 2001); and brain-derived neurotrophic factor (BDNF) may be one link. The less functional variant of a polymorphism in the brain derived-neurotrophic factor gene (*BDNF*), but not the short allele of 5-HTTLPR, has for example been associated with decreased fronto-cingulate activity in response to emotional stimuli in PMDD patients in the luteal phase (Comasco et al., 2014). In line, no 5-HTTLPR genotype effect was observed in the present study.

We found a negative association between allopregnanolone and general alertness, as measured by the Simple Reaction Test. This finding was expected, given the agonistic effects of allopregnanolone on the GABA_A_ receptor (Wang, 2011). In line with a direct effect on the GABA_A_ receptor, we found no evidence that slower reaction times was mediated by lower SERT binding.

## Conclusion

To conclude, considering strengths and limitations of the study, the present findings add knowledge to our understanding of allopregnanolone-SERT neurochemistry in brain regions regulating cognition and emotion processing of potential relevance for sex-specific psychiatric disorders. Further studies with samples from the luteal phase, and also including women with diagnosed PMDD, are needed before a full interpretation of the allopregnanolone-SERT relationship can be made.

## Author Contributions

IS, EC, and VF were involved in the conception and design of the study. TB, MB, and PJ participated in the acquisition of data. IS and EC did the data analyses. VF, TB, MB, and PJ contributed to the interpretation. IS and EC drafted the manuscript. All authors revised the manuscript critically for important intellectual content and approved the final version of the manuscript.

## Conflict of Interest Statement

IS serve occasionally on advisory boards or act as invited speaker at scientific meetings for MSD, Bayer Health Care, and Lundbeck A/S. VF has received honorarium as speaker for H Lundbeck A/S. The remaining authors declare that the research was conducted in the absence of any commercial or financial relationships that could be construed as a potential conflict of interest.

## References

[B1] Arelin K. Mueller K. Barth C. Rekkas P. V. Kratzsch J. Burmann I. (2015). Progesterone mediates brain functional connectivity changes during the menstrual cycle-a pilot resting state MRI study. *Front. Neurosci.* 9:44. 10.3389/fnins.2015.00044 25755630 PMC4337344

[B2] Ashburner J. Friston K. (1997). Multimodal image coregistration and partitioning–a unified framework. *NeuroImage* 6 209–217. 10.1006/nimg.1997.0290 9344825

[B3] Backstrom T. Bixo M. Johansson M. Nyberg S. Ossewaarde L. Ragagnin G. (2014). Allopregnanolone and mood disorders. *Prog. Neurobiol.* 113 88–94. 10.1016/j.pneurobio.2013.07.005 23978486

[B4] Backstrom T. Bixo M. Nyberg S. Savic I. (2012). Increased neurosteroid sensitivity-an explanation to symptoms associated with chronic work related stress in women? *Psychoneuroendocrinology* 38 1078–1089. 10.1016/j.psyneuen.2012.10.014 23177572

[B5] Beliveau V. Svarer C. Frokjaer V. G. Knudsen G. M. Greve D. N. Fisher P. M. (2015). Functional connectivity of the dorsal and median raphe nuclei at rest. *NeuroImage* 116 187–195. 10.1016/j.neuroimage.2015.04.065 25963733 PMC4468016

[B6] Bixo M. Ekberg K. Poromaa I. S. Hirschberg A. L. Jonasson A. F. Andreen L. (2017). Treatment of premenstrual dysphoric disorder with the GABAA receptor modulating steroid antagonist Sepranolone (UC1010)-A randomized controlled trial. *Psychoneuroendocrinology* 80 46–55. 10.1016/j.psyneuen.2017.02.031 28319848

[B7] Brunton P. J. (2015). Programming the brain and behaviour by early-life stress: a focus on neuroactive steroids. *J. Neuroendocrinol.* 27 468–480. 10.1111/jne.12265 25688636

[B8] Comasco E. Hahn A. Ganger S. Gingnell M. Bannbers E. Oreland L. (2014). Emotional fronto-cingulate cortex activation and brain derived neurotrophic factor polymorphism in premenstrual dysphoric disorder. *Hum. Brain Mapp.* 35 4450–4458. 10.1002/hbm.22486 24615932 PMC4107029

[B9] Comasco E. Sundstrom-Poromaa I. (2015). Neuroimaging the menstrual cycle and premenstrual dysphoric disorder. *Curr. Psychiatry Rep.* 17:77. 10.1007/s11920-015-0619-4 26272540

[B10] Derogatis L. R. Savitz K. L. (1999). “The SCL-90-R, Brief Symptom Inventory, and Matching Clinical Rating Scales,” in *The use of Psychological Testing for Treatment Planning and Outcomes Assessment*, ed. Maruish M. E. (Mahwah, NJ: Lawrence Erlbaum Associates), 679–724.

[B11] Devall A. J. Santos J. M. Fry J. P. Honour J. W. Brandao M. L. Lovick T. A. (2015). Elevation of brain allopregnanolone rather than 5-HT release by short term, low dose fluoxetine treatment prevents the estrous cycle-linked increase in stress sensitivity in female rats. *Eur. Neuropsychopharmacol.* 25 113–123. 10.1016/j.euroneuro.2014.11.017 25498416

[B12] Erritzoe D. Holst K. Frokjaer V. G. Licht C. L. Kalbitzer J. Nielsen F. A. (2010). A nonlinear relationship between cerebral serotonin transporter and 5-HT(2A) receptor binding: an in vivo molecular imaging study in humans. *J. Neurosci.* 30 3391–3397. 10.1523/JNEUROSCI.2852-09.2010 20203198 PMC6634111

[B13] Fisher P. M. Larsen C. B. Beliveau V. Henningsson S. Pinborg A. Holst K. K. (2017a). Pharmacologically induced sex hormone fluctuation effects on resting-state functional connectivity in a risk model for depression: a randomized trial. *Neuropsychopharmacology* 42 446–453. 10.1038/npp.2016.208 27649641 PMC5399242

[B14] Fisher P. M. Ozenne B. Svarer C. Adamsen D. Lehel S. Baare W. F. (2017b). BDNF val66met association with serotonin transporter binding in healthy humans. *Transl. Psychiatry* 7:e1029. 10.1038/tp.2016.295 28195567 PMC5438027

[B15] Frokjaer V. G. Pinborg A. Holst K. K. Overgaard A. Henningsson S. Heede M. (2015). Role of serotonin transporter changes in depressive responses to sex-steroid hormone manipulation: a positron emission tomography study. *Biol. Psychiatry* 78 534–543. 10.1016/j.biopsych.2015.04.015 26004162

[B16] Frokjaer V. G. Vinberg M. Erritzoe D. Svarer C. Baare W. Budtz-Joergensen E. (2009). High familial risk for mood disorder is associated with low dorsolateral prefrontal cortex serotonin transporter binding. *NeuroImage* 46 360–366. 10.1016/j.neuroimage.2009.02.008 19233297

[B17] Fry J. P. Li K. Y. Devall A. J. Cockcroft S. Honour J. W. Lovick T. A. (2014). Fluoxetine elevates allopregnanolone in female rat brain but inhibits a steroid microsomal dehydrogenase rather than activating an aldo-keto reductase. *Br. J. Pharmacol.* 171 5870–5880. 10.1111/bph.12891 25161074 PMC4290723

[B18] Griffin L. D. Mellon S. H. (1999). Selective serotonin reuptake inhibitors directly alter activity of neurosteroidogenic enzymes. *Proc. Natl. Acad. Sci. U.S.A.* 96 13512–13517. 10.1073/pnas.96.23.13512 10557352 PMC23979

[B19] Haahr M. E. Hansen D. L. Fisher P. M. Svarer C. Stenbaek D. S. Madsen K. (2015). Central 5-HT neurotransmission modulates weight loss following gastric bypass surgery in obese individuals. *J. Neurosci.* 35 5884–5889. 10.1523/JNEUROSCI.3348-14.2015 25855196 PMC6605315

[B20] Heils A. Teufel A. Petri S. Stober G. Riederer P. Bengel D. (1996). Allelic variation of human serotonin transporter gene expression. *J. Neurochem.* 66 2621–2624. 10.1046/j.1471-4159.1996.66062621.x 8632190

[B21] Hellgren C. Akerud H. Skalkidou A. Backstrom T. Sundstrom-Poromaa I. (2014). Low serum allopregnanolone is associated with symptoms of depression in late pregnancy. *Neuropsychobiology* 69 147–153. 10.1159/000358838 24776841

[B22] Hellgren C. Comasco E. Skalkidou A. Sundstrom-Poromaa I. (2017). Allopregnanolone levels and depressive symptoms during pregnancy in relation to single nucleotide polymorphisms in the allopregnanolone synthesis pathway. *Horm. Behav.* 94 106–113. 10.1016/j.yhbeh.2017.06.008 28666923

[B23] Ichise M. Liow J. S. Lu J. Q. Takano A. Model K. Toyama H. (2003). Linearized reference tissue parametric imaging methods: application to [^11^C]DASB positron emission tomography studies of the serotonin transporter in human brain. *J. Cereb. Blood Flow Metab.* 23 1096–1112. 10.1097/01.WCB.0000085441.37552.CA 12973026

[B24] Kalbitzer J. Erritzoe D. Holst K. K. Nielsen F. A. Marner L. Lehel S. (2010). Seasonal changes in brain serotonin transporter binding in short serotonin transporter linked polymorphic region-allele carriers but not in long-allele homozygotes. *Biol. Psychiatry* 67 1033–1039. 10.1016/j.biopsych.2009.11.027 20110086

[B25] Kanes S. Colquhoun H. Gunduz-Bruce H. Raines S. Arnold R. Schacterle A. (2017). Brexanolone (SAGE-547 injection) in post-partum depression: a randomised controlled trial. *Lancet* 390 480–489. 10.1016/S0140-6736(17)31264-3 28619476

[B26] Kask K. Backstrom T. Nilsson L. G. Sundstrom-Poromaa I. (2008). Allopregnanolone impairs episodic memory in healthy women. *Psychopharmacology* 199 161–168. 10.1007/s00213-008-1150-7 18551282

[B27] Knudsen G. M. Jensen P. S. Erritzoe D. Baare W. F. Ettrup A. Fisher P. M. (2016). The center for integrated molecular brain imaging (Cimbi) database. *NeuroImage* 124 1213–1219. 10.1016/j.neuroimage.2015.04.025 25891375

[B28] Lesch K. P. Bengel D. Heils A. Sabol S. Z. Greenberg B. D. Petri S. (1996). Association of anxiety-related traits with a polymorphism in the serotonin transporter gene regulatory region. *Science* 274 1527–1531. 10.1126/science.274.5292.1527 8929413

[B29] Macoveanu J. Henningsson S. Pinborg A. Jensen P. Knudsen G. M. Frokjaer V. G. (2016). Sex-steroid hormone manipulation reduces brain response to reward. *Neuropsychopharmacology* 41 1057–1065. 10.1038/npp.2015.236 26245498 PMC4748430

[B30] Melcangi R. C. Panzica G. Garcia-Segura L. M. (2011). Neuroactive steroids: focus on human brain. *Neuroscience* 191 1–5. 10.1016/j.neuroscience.2011.06.024 21704130

[B31] Mengod G. Palacios J. M. Cortes R. (2015). Cartography of 5-HT1A and 5-HT2A receptor subtypes in prefrontal cortex and its projections. *ACS Chem. Neurosci.* 6 1089–1098. 10.1021/acschemneuro.5b00023 25739427

[B32] Moses E. L. Drevets W. C. Smith G. Mathis C. A. Kalro B. N. Butters M. A. (2000). Effects of estradiol and progesterone administration on human serotonin 2A receptor binding: a PET study. *Biol. Psychiatry* 48 854–860. 10.1016/S0006-3223(00)00967-7 11063980

[B33] Moses-Kolko E. L. Berga S. L. Greer P. J. Smith G. Cidis Meltzer C. Drevets W. C. (2003). Widespread increases of cortical serotonin type 2A receptor availability after hormone therapy in euthymic postmenopausal women. *Fertil. Steril.* 80 554–559. 10.1016/S0015-0282(03)00973-7 12969697

[B34] Moses-Kolko E. L. Price J. C. Shah N. Berga S. Sereika S. M. Fisher P. M. (2011). Age, sex, and reproductive hormone effects on brain serotonin-1A and serotonin-2A receptor binding in a healthy population. *Neuropsychopharmacology* 36 2729–2740. 10.1038/npp.2011.163 21849982 PMC3230496

[B35] Newberg A. B. Amsterdam J. D. Wintering N. Shults J. (2012). Low brain serotonin transporter binding in major depressive disorder. *Psychiatry Res.* 202 161–167. 10.1016/j.pscychresns.2011.12.015 22698760 PMC3398160

[B36] Nyberg S. Backstrom T. Zingmark E. Purdy R. H. Poromaa I. S. (2007). Allopregnanolone decrease with symptom improvement during placebo and gonadotropin-releasing hormone agonist treatment in women with severe premenstrual syndrome. *Gynecol. Endocrinol.* 23 257–266. 10.1080/09513590701253511 17558683

[B37] Ottander U. Poromaa I. S. Bjurulf E. Skytt A. Backstrom T. Olofsson J. I. (2005). Allopregnanolone and pregnanolone are produced by the human corpus luteum. *Mol. Cell. Endocrinol.* 239 37–44. 10.1016/j.mce.2005.04.007 15935549

[B38] Parsey R. V. Hastings R. S. Oquendo M. A. Huang Y. Y. Simpson N. Arcement J. (2006). Lower serotonin transporter binding potential in the human brain during major depressive episodes. *Am. J. Psychiatry* 163 52–58. 10.1176/appi.ajp.163.1.52 16390889

[B39] Pinna G. Costa E. Guidotti A. (2009). SSRIs act as selective brain steroidogenic stimulants (SBSSs) at low doses that are inactive on 5-HT reuptake. *Curr. Opin. Pharmacol.* 9 24–30. 10.1016/j.coph.2008.12.006 19157982 PMC2670606

[B40] Schule C. Nothdurfter C. Rupprecht R. (2014). The role of allopregnanolone in depression and anxiety. *Prog. Neurobiol.* 113 79–87. 10.1016/j.pneurobio.2013.09.003 24215796

[B41] Segebladh B. Borgstrom A. Nyberg S. Bixo M. Sundstrom-Poromaa I. (2009). Evaluation of different add-back estradiol and progesterone treatments to gonadotropin-releasing hormone agonist treatment in patients with premenstrual dysphoric disorder. *Am. J. Obstet. Gynecol.* 201 e131–e138. 10.1016/j.ajog.2009.03.016 19398092

[B42] Stein P. Baldinger P. Kaufmann U. Christina R. M. Hahn A. Hoflich A. (2014). Relation of progesterone and DHEAS serum levels to 5-HT1A receptor binding potential in pre- and postmenopausal women. *Psychoneuroendocrinology* 46 52–63. 10.1016/j.psyneuen.2014.04.008 24882158

[B43] Stenbaek D. S. Fisher P. M. Budtz-Jorgensen E. Pinborg A. Hjordt L. V. Jensen P. S. (2016). Sex hormone manipulation slows reaction time and increases labile mood in healthy women. *Psychoneuroendocrinology* 68 39–46. 10.1016/j.psyneuen.2016.02.023 26943343

[B44] Sundstrom I. Nyberg S. Bixo M. Hammarback S. Backstrom T. (1999). Treatment of premenstrual syndrome with gonadotropin-releasing hormone agonist in a low dose regimen. *Acta Obstet. Gynecol. Scand.* 78 891–899. 10.1080/j.1600-0412.1999.781011.x 10577620

[B45] Sundstrom Poromaa I. Gingnell M. (2014). Menstrual cycle influence on cognitive function and emotion processing-from a reproductive perspective. *Front. Neurosci.* 8:380. 10.3389/fnins.2014.00380 25505380 PMC4241821

[B46] Svarer C. Madsen K. Hasselbalch S. G. Pinborg L. H. Haugbol S. Frokjaer V. G. (2005). MR-based automatic delineation of volumes of interest in human brain PET images using probability maps. *NeuroImage* 24 969–979. 10.1016/j.neuroimage.2004.10.017 15670674

[B47] Svensson A. I. Berntsson A. Engel J. A. Soderpalm B. (2000). Disinhibitory behavior and GABA(A) receptor function in serotonin-depleted adult male rats are reduced by gonadectomy. *Pharmacol. Biochem. Behav.* 67 613–620. 10.1016/S0091-3057(00)00403-2 11164093

[B48] Syan S. K. Minuzzi L. Costescu D. Smith M. Allega O. R. Coote M. (2017). Influence of endogenous estradiol, progesterone, allopregnanolone, and dehydroepiandrosterone sulfate on brain resting state functional connectivity across the menstrual cycle. *Fertil. Steril.* 107:e1244. 10.1016/j.fertnstert.2017.03.021 28476183

[B49] Timby E. Balgard M. Nyberg S. Spigset O. Andersson A. Porankiewicz-Asplund J. (2006). Pharmacokinetic and behavioral effects of allopregnanolone in healthy women. *Psychopharmacology* 186 414–424. 10.1007/s00213-005-0148-7 16177884

[B50] Toffoletto S. Lanzenberger R. Gingnell M. Sundstrom-Poromaa I. Comasco E. (2014). Emotional and cognitive functional imaging of estrogen and progesterone effects in the female human brain: a systematic review. *Psychoneuroendocrinology* 50 28–52. 10.1016/j.psyneuen.2014.07.025 25222701

[B51] Tyrer A. E. Levitan R. D. Houle S. Wilson A. A. Nobrega J. N. Meyer J. H. (2016). Increased seasonal variation in serotonin transporter binding in seasonal affective disorder. *Neuropsychopharmacology* 41 2447–2454. 10.1038/npp.2016.54 27087270 PMC4987850

[B52] Uzunov D. P. Cooper T. B. Costa E. Guidotti A. (1996). Fluoxetine-elicited changes in brain neurosteroid content measured by negative ion mass fragmentography. *Proc. Natl. Acad. Sci. U.S.A.* 93 12599–12604. 10.1073/pnas.93.22.12599 8901628 PMC38038

[B53] van Wingen G. A. van Broekhoven F. Verkes R. J. Petersson K. M. Backstrom T. Buitelaar J. K. (2008). Progesterone selectively increases amygdala reactivity in women. *Mol. Psychiatry* 13 325–333. 10.1038/sj.mp.4002030 17579609

[B54] Wang M. (2011). Neurosteroids and GABA-A receptor function. *Front. Endocrinol.* 2:44 10.3389/fendo.2011.00044 PMC3356040 22654809

[B55] Whitaker-Azmitia P. M. (2001). Serotonin and brain development: role in human developmental diseases. *Brain Res. Bull.* 56 479–485. 10.1016/S0361-9230(01)00615-3 11750793

[B56] Woods R. P. Cherry S. R. Mazziotta J. C. (1992). Rapid automated algorithm for aligning and reslicing PET images. *J. Comput. Assist. Tomogr.* 16 620–633. 10.1097/00004728-199207000-00024 1629424

[B57] Yan Z. (2002). Regulation of GABAergic inhibition by serotonin signaling in prefrontal cortex: molecular mechanisms and functional implications. *Mol. Neurobiol.* 26 203–216. 10.1385/MN:26:2-3:203 12428756

